# Synthetic Approaches for C-N Bonds by TiO_2_ Photocatalysis

**DOI:** 10.3389/fchem.2019.00635

**Published:** 2019-09-18

**Authors:** Dongge Ma, Shan Zhai, Yi Wang, Anan Liu, Chuncheng Chen

**Affiliations:** ^1^Key Laboratory of Cosmetic of China National Light Industry, School of Science, Beijing Technology and Business University, Beijing, China; ^2^Basic Experimental Center for Natural Science, University of Science and Technology Beijing, Beijing, China; ^3^Key Laboratory of Photochemistry, Beijing National Laboratory for Molecular Sciences, Institute of Chemistry, Chinese Academy of Sciences, Beijing, China

**Keywords:** TiO_2_, heterogeneous photocatalysis, C-N bond formation, amine, heterocycle

## Abstract

Nitrogen-containing organic compounds possess the most important status in drug molecules and agricultural chemicals. More than 80% currently used drugs have at least a C-N bond. The green and mild methodology to prepare diverse C-N bonds to replace traditional harsh preparation protocols is always a hotspot in modern synthetic chemistry. TiO_2_-based nanomaterials, considered as environmentally benign, stable, and powerful photocatalysts, have recently been applied in some certain challenging organic synthesis including construction of useful C-N compounds under mild conditions that are impossible to complete by conventional catalysis. This mini review would present state-of-the-art paragon examples of TiO_2_ photocatalyzed C-N bond formations. The discussion would be divided into two main sections: (1) N-alkylation of amines and (2) C-N formation in heterocycle synthesis. Especially, the mechanism of TiO_2_ photocatalytic C-N bond formation through activating alcohol into C=O by photo-induced hole followed by C=NH-R formation and finally hydrogenating C=NH-R into C-N bonds by combination of photo-induced electron/H^+^ assisted with loaded-Pt would be covered in detail. We believe that the mini-review will bring new insights into TiO_2_ photocatalysis applied to construct challenging organic compounds through enabling photo-induced hole and electron in a concerted way on coupling two substrate molecules together with respect to their conventionally independent catalysis behavior.

## Introduction

**Figure 1:**
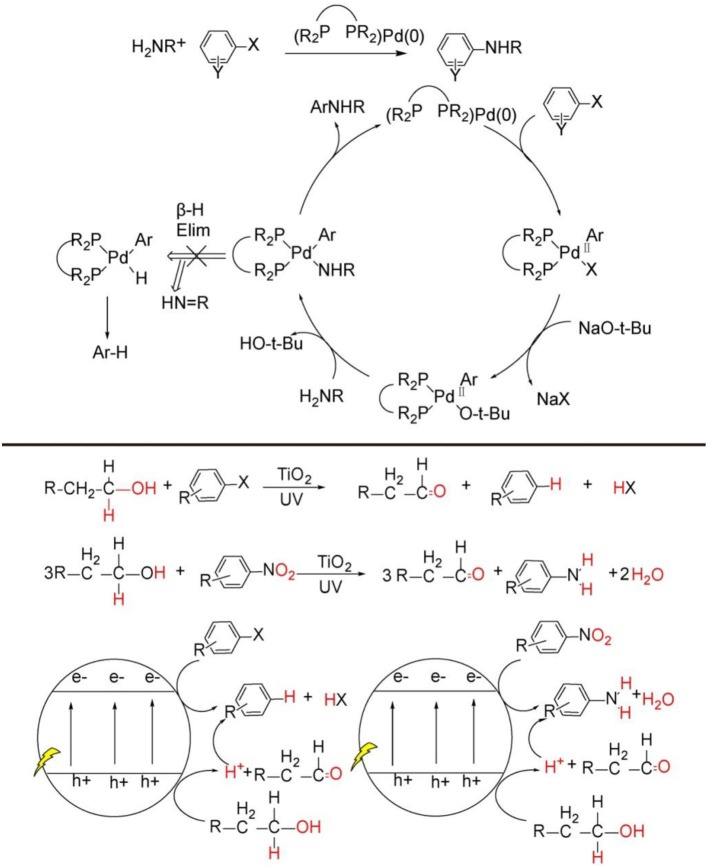
C-N bond construction through transition-metal catalytic strategy (**Top**), general pathway of organic synthesis mediated by TiO_2_ photocatalysis (**Bottom**).

Nitrogen is among the most ubiquitous elements in the nature. The nitrogen-containing organic structure unit constitutes the basic building block of life, such as proteins, DNA, and RNA. Moreover, most natural products, pharmaceuticals and agrochemicals demand nitrogen-containing group for their particular activity (Taylor et al., 2014). Nowadays, a number of catalytic methods have been exploited to construct diverse functional C-N bonds. Among them, palladium catalyzed Buchwald-Hartwig reaction (Bruno et al., 2013; Ruiz-Castillo and Buchwald, 2016; Heravi et al., 2018) and copper catalyzed Ullmann reaction (Beletskaya and Cheprakov, 2012) and Chan-Lam coupling (Fischer and Koenig, 2011; Qiao and Lam, 2011; Duan et al., 2019) are the first choices due to their high efficiency, excellent chemo-, regioselectivity, and yields (see Figure 1, top). Despite their widespread uses, these transition-metal catalyzed methods have some intrinsic disadvantages. For example, the reactions usually proceed under obligatory high refluxing temperature, rigorous, and complicated anaerobic and anhydrous manipulation; the catalysts generally demand complex and expensive ligands; there are non-trivial separations of homogeneous catalysts; the bio-toxicity of the transition-metal catalyst easily remain in the final product. What is more, these catalytic strategies require strictly pre-functionalized substrates because common C-H compounds do not quite meet the request of the target C-N bonds synthesis. All of these issues call for alternative catalytic approaches (Santoro et al., 2016; Ma et al., 2018b) directly activating C-H bonds of more common compounds to construct diverse C-N bonds.

In recent years, heterogeneous photoredox synthesis has experienced a renaissance with the emergence of new and highly active photocatalysts (Kisch, 2013, 2017; Friedmann et al., 2016; Parrino et al., 2018). Among the heterogeneous semiconductor photocatalysts, TiO_2_ nanoparticle, as the most explored one, is prevalently investigated because it possesses very powerful photo-generated hole on valance band and electrons on conduction band, by which most inert C-H bonds can be readily activated for the synthesis of value-added organic products by TiO_2_ or metal-loaded/TiO_2_ photocatalysis (see Figure 1, bottom) (Kuntz, 1997; Yurdakal et al., 2008; Zhang et al., 2008, 2014; Higashimoto et al., 2009; Füldner et al., 2010; Kohtani et al., 2010, 2018; Zhu et al., 2010; Palmisano et al., 2011; Cherevatskaya and Koenig, 2014; Manley et al., 2014; Hoffmann, 2015; Lang et al., 2015a,b; Manley and Walton, 2015; Ma et al., 2017, 2018a, 2019; Ma and Li, 2018; Wang Y. et al., 2018;). However, these previous TiO_2_ photocatalysis examples for synthetic applications mainly activate the substrates separately on photo-induce valence band and conduction band, and the products are either oxidative or reductive products (see Figure 1, bottom). There are very rare reports focused on coupling photo-induced holes and electrons synergistically to realize more significant cross-coupling reactions with wider substrate scopes and excellent functional group tolerability.

**Figure 2:**
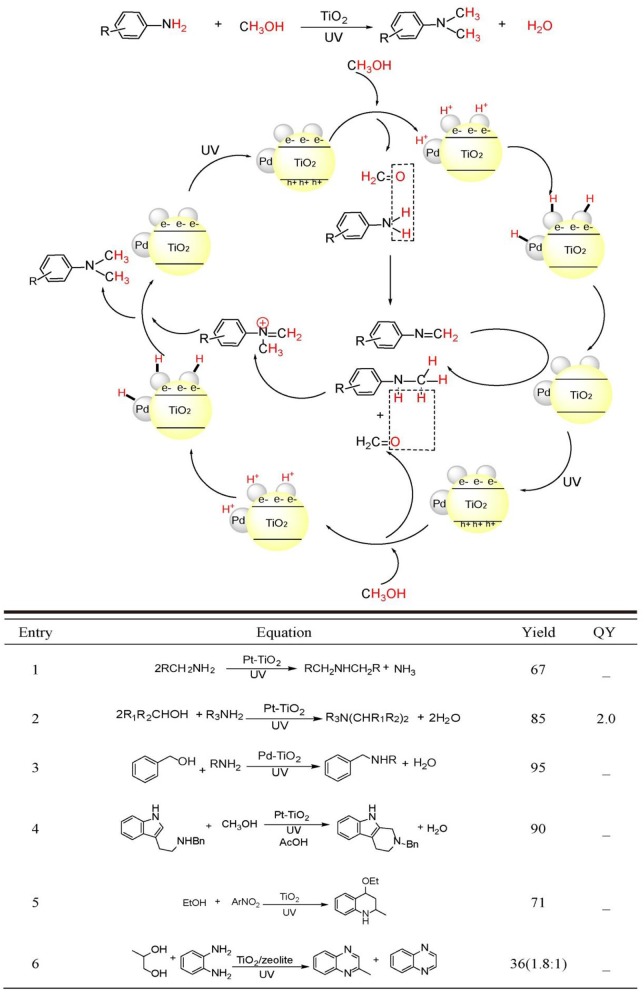
C-N bond constructions by TiO_2_ photocatalysis (**Top**) two kinds of C-N bond formation catalyzed by TiO_2_ photocatalysis: (Equations 1–4) N-alkylation and (Equations 5, 6) N-heterocyclization using alcohols as starting reagents. Equations (1)–(6) see details in the following main text (**Bottom**).

Very recently, TiO_2_ photocatalysis is developed to successfully tune photo-induced hole/electron pair synergistically for synthetically important coupling reactions such as C-C bonds formation (Manley et al., 2012; Rueping et al., 2012; Ma et al., 2015; Liu et al., 2016; Nauth et al., 2018) and C-N bond formations (Vila and Rueping, 2013) (see Figure 1, bottom). These examples evidently endowed TiO_2_ photocatalysis prominent perspective for organic synthetic applications. Since TiO_2_ photocatalyzed oxidation, reduction, and C-C formation have been extensively reviewed (Palmisano et al., 2007, 2010; Ravelli et al., 2011; Augugliaro et al., 2015), this mini-review mainly focus on the TiO_2_ photocatalyzed useful C-N bond formations. We will divide the discussion into two sections: N-alkylation of amines (see Figure 2, bottom Equations 1–4) and C-N formation in heterocycle synthesis (see Figure 2, bottom Equations 5–6).

Why C-N bonds can be formed by TiO_2_ photocatalyst in these two kinds of reactions? The main reason is that in these two C-N bond formation reactions, the photo-induced hole oxidation and electron reduction are highly coupled by an intermediate. For the case of N-alkylation of amines (see Figure 2, top), TiO_2_ photocatalysis uses and activates environmentally more friendly C-H and -OH bond of alcohol -CH_2_-OH into -HC=O bond intermediates by photo-induce hole powerful oxidation, which readily reacts with R-NH_2_ substrates into -HC=N-R even without catalysis. Finally, the as-formed key C=N-R intermediates are hydrogenated into –CH_2_-NH-R by conduction band electron coupled with H^+^ assisted by loaded Pt. Such an imine intermediate formation is much easier to yield C-N bond than that of two transmetalation steps (R_1_C-Pd(II)-X_2_Lx to R_1_C-Pd(II)-O-^t^BuLx → R_1_C-Pd(II)-NR_2_Lx) in transition-metal catalysis(see Figure 1, top). For the case of C-N formation in heterocycle synthesis, unlike the above-mentioned pathway that valence-band hole and conduction-band electron both act on the same substrate and the other substrate R_2_NH involves in coupling reaction with alcohol photo-oxidized product aldehyde without photocatalysis, this kind of reaction depends on photo-induced holes and electrons separately activates two substrates by two-electron-transfer. And the two separate intermediates implements double condensation of -HC=O and R-NH_2_ and provide the final cyclization product. These two C-N bond formation reactions both require valence-band holes and conduction-band electrons synergistic interaction. Moreover, the formation of a long life-time intermediate –C=NR is the prerequisite for the sufficient probability to couple conduction-band electron/H^+^ for target product. The previously reported TiO_2_ photocatalysis could not realize A + B → C type coupling reaction, because it lacks the suitable long life-time stable intermediate to accommodate and tune the synergistic interaction of valence-band hole and conduction-band electron. In the following sections, we will introduce and comment on these two typical kinds of TiO_2_ photocatalytic C-N synthesis in detail.

## TiO_2_ Photocatalyzed Amine N-alkylation

N-alkylated amines are very important nitrogen-containing compounds in pharmaceuticals, agrochemicals, dyes, and functional materials. The traditional synthetic routes for N-alkylated amines can be divided into two categories: (i) reductive aminations using carbonyls, amines, and stoichiometric metal hydride reductants (Abdel-Magid and Mehrman, 2006); (ii) transition-metal catalyzed substitution of amines with alkyl or aryl halides or pseudo halides (Shin et al., 2015). Although, these mature methodologies are viable and provide excellent yields for most amines, they still suffer from some drawbacks such as the bio-toxicity of aldehydes, organic halides, and transition-metal catalyst, the harsh operating conditions such as high temperature and the use of hazardous metal hydrides as reductants. The high temperature above 100°C can be very detrimental for the late-stage functionalization of unstable and oxidable primary amines. To meet these demands, using a greener alkylating reagent and conducting the reaction in milder conditions are highly desirable. TiO_2_ photocatalysis using low-toxic alcohols as alkylating reagents can be an excellent choice to solve this issue. This success is largely ascribed to both powerful potential of photo-generated hole in initiating more inert but less toxic substrates and hole-electrons as traceless reagents in the final products.

Dating back to 1983, Kagiya et al. reported that primary alkyl amine could be photo-dimerized to yield secondary dialkyl amine with the loss of NH_3_ by Pt/TiO_2_ photocatalyst in aqueous suspension (Equation 1) (Nishimoto et al., 1983). The authors proposed that TiO_2_ photo-induced valence-band hole was responsible for the oxidation of primary amine to primary iminium ion. The nucleophilic attack of the remaining amine to iminium ion generated the coupled secondary imine, while the photo-induced conduction-band electrons reduced aqueous proton and combined with Pt nanoparticle generating Pt-H species. The Pt-H species reduced secondary imine to the target dialkyl amine. This work inaugurated the application of TiO_2_ photocatalysis to C-N coupling reactions.

Using alcohols other than aliphatic amines as alkylating reagents was proven to be a more efficient strategy with less side reactions for the Pt/TiO_2_ photocatalyzed amine N-alkylation because alcohols are more stable than aliphatic amines under UV-light irradiation. Kagiya et al. reported that unsymmetrical secondary amine or tertiary amine could be synthesized by the photocatalytic cross-coupling between amine and alcohol (Ohtani et al., 1986, 1990) (Equation 2). In an alcoholic solution, primary amine could be transformed to the N-alkylated product by Pt/TiO_2_ photocatalysis. Prolonged irradiation leaded the formation of N,N'-dialkylated products. Cyclic and acyclic secondary amine participated in N-alkylation providing tertiary amine. The authors proposed that the amine product was initially transformed to iminium ion as in the previous examples. Photo-induced electrons reduced alcoholic proton yielding H_2_. H_2_ adsorbed on Pt nanoparticles performed the *in-situ* reduction of secondary imine to N-alkylated secondary amines. The substitution of N-alkylating reagent and solvent from amine and H_2_O to alcohol greatly enhanced the yield. This mainly originated to the more facile loss of H_2_O compared with NH_3_. On the other hand, reduction of proton in alcohol was extremely easier than proton in H_2_O.

Although, amine N-alkylation by Pt/TiO_2_ photocatalysis experienced success in 1980s, the issue on the control of chemoselectivity between N-monoalkylation and N,N'-dialkylation had no practicability by using this catalyst system. Shiraishi et al. solved this issue by the utilization of Pd/TiO_2_ photocatalyst other than Pt/TiO_2_. (Shiraishi et al., 2013) (Equation 3) Loading Pd nanoparticles with 2–2.5 nm diameter onto TiO_2_ surface can achieve the highest yield of N-monoalkylation product. This catalyst promoted the rate-determining-step, i.e., imine hydrogenation utmost. The secret to obtain the N-monoalkylation products with high chemoselectivity was the control of suitable irradiation time and the application of sterically hindered substrates.

Apart from Pt/TiO_2_ and Pd/TiO_2_, Au/TiO_2_ also photocatalyzed the N-alkylation of aniline using alcohols as alkylating reagents (Stibal et al., 2013). Moreover, one-pot tandem synthesis of N-alkylated aniline can be achieved from nitrobenzene substrates by this method. The authors observed dialkylated product only when methanol was used as alkylating reagent. Using other chain alcohols all provided N-monoalkylated products. The lowest pKa conferred methanol with the highest reactivity to form the dialkylated products.

Compared with Pt, Pd, and Au/TiO_2_ photocatalyst, Ag/TiO_2_ evidenced its power in wider substrates scope and better functional group tolerability. Saito et al. reported that under UV irradiation, Ag/TiO_2_ photocatalyst can initiate N-methylation to amines with various functional groups intact (Tsarev et al., 2015). Amines possessing N-benzyl, N-allyl, N-Boc, hydroxyl, ether, acetal, carboxamide, formamide, and olefin groups were all well-tolerated under this mild photoreaction conditions. Moreover, this method can be used to the methylation of chiral amine with almost complete chirality retention. Otherwise, NH_3_ and proline N-methylation can be successfully accomplished in aqueous solution with unreduced yields. Besides, this method has very high selectivity to amine substrates in the presence of various other reducible compounds.

Besides intermolecular N-alkylation, intramolecular N-alkylation can also be realized using Pt/TiO_2_ photocatalyst. Uyeda et al. reported that 2,3,4,9-tetrahydro-1H-carbazoles can be synthesized via a photocatalytic Pictet-Spengler reaction from 2-(1H-indol-3-yl)ethan-1-amine and methanol (Adolph et al., 2017) (Equation 4). They discovered that an intermolecular N-methylation between methanol and 2-(1H-indol-3-yl) ethan-1-amine can be coupled with the intramolecular C-C bond formation on indole 2-position and N-methyl group. Only Pt/TiO_2_ could provide the target cyclization carbazole product, while other metals such as Au, Ag, or Pd loaded TiO_2_ all did not realize this transformation. Ag and Au could not effectively convert the tryptamine substrate. Pd/TiO_2_ had the power to completely consume the reactant. However, N-methylation other than cyclization product was generated under this condition. A wide substrate scope was explored and various functional groups were tolerated with this method. Moreover, this photocatalysis system could be applied to other intermolecular multicomponent reactions such as intermolecular addition, Strecker reaction, Mannich, and Ugi-type reaction with good to excellent yields.

Cu/TiO_2_ and Au/TiO_2_ mixed photocatalyst could alkylate the complex functionalized aromatic amines (Wang L.-M. et al., 2018). In this composite catalyst, Au/TiO_2_ moiety was responsible for dehydrogenation of alcohol to form aldehyde *in-situ*, while Cu/TiO_2_ moiety catalyzed the reduction of imine intermediate to the final N-alkylated amine product. This report was the first example applying mixed metal/TiO_2_ based photocatalyst for N-alkylation of complex functionalized amines.

Although, TiO_2_ photocatalysis has accumulated a plethora of delicate examples for amine N-alkylation to complement traditional reductive amination and transition-metal catalyzed processes, there are still much spaces and gaps for the researcher in this area to surpass. Firstly, developing more low-cost non-noble-metal loaded TiO_2_ photocatalyst systems to achieve the same or higher yields and chemoselectivity for amine N-alkylation are strongly demanded. Second, if visible-light active TiO_2_ photocatalyst systems can be applied in this transformation, the solar energy utilization efficiency would be greatly enhanced since the current TiO_2_ and metal-loaded TiO_2_ nanoparticle systems can only capture the UV light, which covers only 5% energy in sun spectrum. Last but not the least, enhancing the chemoselectivity of more complex target molecules with accurate tuning between N-monoalkylation and N,N'-dialkylation is extremely pivotal for the further development in this field. Especially, due to the more and more rigorous demanding in chiral pure pharmaceuticals, realizing asymmetric N-alkylation by TiO_2_ heterogeneous photocatalyst systems would be the ultimate goal. In order to realize this, fabricating diverse asymmetric TiO_2_ surfaces may be the ideal choice compared with previously reported adding molecular chiral co-catalyst into the suspension. If all the above-mentioned limitations were perfectly resolved, in light of its powerful potential to activate nearly any inert C-H compounds, this method using TiO_2_ heterogeneous photocatalyst would possess the comparable status to cover the shortages for transition-metal catalysis, which commonly requires highly functionalized substrates.

## TiO_2_ Photocatalyzed N-heterocycle Formation

N-heterocycles are the most important molecular scaffold for life. A lot of natural products comprised N-heterocycles. Moreover, the report provided by FDA demonstrated that about 60% pharmaceutical molecules contain N-heterocycles (Newman and Cragg, 2016). Although, there are a number of methodologies to prepare N-heterocycles *de novo* by transition-metal (Shaikh and Hong, 2016) or acid/base catalysis (Doustkhah et al., 2019), developing much greener and economic methods without uses of unavailable starting materials or pre-functionalized substrates are urgently desired. TiO_2_ photocatalysts are deemed to be ideal choice for this task because of their powerful ability to activate most inert C-H, C-C, and C-X bonds of common substrates and the environmentally benign properties.

As early as 1990s, Park et al. firstly realized the N-heterocycle formation by TiO_2_ photocatalysis (Park et al., 1995) (Equation 5). Upon UV irradiation, TiO_2_ nanoparticles catalyzed the synthesis of 4-ethoxy-l,2,3,4-tetrahydroquinoline from nitroarene and ethanol in a one-pot process, in which ethanol was transformed sequentially to acetaldehyde and ethyl vinyl ether, while nitroarene was transformed to Schiff base by a reductive amination process. The final hetero-Diers-Alder cycloaddition furnished the target tetrahydroquinoline product with ~71% yield.

Under aerobic conditions, TiO_2_/zeolite photocatalyst system furnished 2-methyl quinoxaline and quinoxaline from o-phenyldiamine and propyleneglycol with 22.5 and 12.6% yield, respectively (Rao and Subrahmanyam, 2002) (Equation 6). The proposed mechanism included three steps: (1) TiO_2_ photocatalyzed oxidation of propyleneglycol to 2-oxopropanal using photo-induced valence-band holes. And the corresponding conduction-band electrons were consumed by O_2_. The *in-situ* generated 2-oxopropanal condensed with o-phenyldiamine yielding 2-methyl quinoxaline. Further photo-oxidation of 2-methyl quinoxaline by photo-induced holes yielded the radical cation, which was transformed to methyl radical by a proton-coupled-electron transfer process by zeolite. The sequential oxidation and decarboxylation steps generated quinoxaline product. The introduction of unselective dioxygen and secondary reactive oxygen species may be the reason for the lower yield.

By the combinative use of TiO_2_ photocatalyst with p-toluenesulfonic acid as a co-catalyst, quinolines could be synthesized from nitrobenzenes (Hakki et al., 2009, 2013). Based on the GC-MS chromatograms analysis of the intermediates, the authors deduced a different reaction pathway that the condensation between two Schiff-bases and acetaldehyde and the sequential cyclization and dehydration facilitated quinoline products other than the conventional crotonaldehyde route. The detection of N-ethyl-3,5-dimethylbenzenamine and the absence of crotonaldehyde in the GC-MS tracing analysis during the photo-reaction evidenced this proposition. Moreover, the same group demonstrated that acid-modified mesoporous SiO_2_ decorated with TiO_2_ could also realize this transformation in anaerobic alcoholic solution yielding poly-substituted quinolines.

## Conclusion

We have conducted a thorough review of the paragon examples of TiO_2_ photocatalyzed C-N formations. Although, still in its infantile period compared with transition-metal catalysis and organocatalysis, TiO_2_ photocatalysis has demonstrated its power for a number of concrete examples to construct C-N bonds using alcohols as mild and green alkylating reagents for amine N-alkylation and the *de novo* synthesis of five- or six-membered N-heterocycles. Such a catalytic strategy stems from the powerful photo-generated holes/electrons on TiO_2_ nanoparticle surfaces, which readily activates inert C-H, C-C, and C-X of common organic compounds to construct useful C-N compounds. Figure 2, bottom summarized the yields and quantum yield of C-N compounds obtained by these TiO_2_-based photocatalysts. Compared with a number of various visible-light responsive photocatalyst materials, TiO_2_ possesses higher valence-band hole oxidative ability (E_vb_ = 2.7 V vs. NHE at pH = 7). This confers it more possibility for activation of organic compounds inert bonds such as C-H and C-X. So, the dehydrogenation process of C-OH to C=O on TiO_2_ photocatalysis proceeds facilely. However, due to the moderate reductive ability of its conduction-band (E_cb_ = −0.5 V vs. NHE at pH = 7), realizing C-N single bond formation usually requires the noble-metal assistance. Some visible-light responsive nanomaterials including Au, Pd nanoparticles, metal-organic frameworks, CdS, ZnIn_2_S_4_, and BiVO_4_, these photocatalysts has narrower band-gap, but lower ability to activate inert bonds. Most of these photocatalysts were applied in the aerobic oxidation of amine to imine. In these transformations, dioxygen other than C=N intermediates acts as the final electron acceptor. Apart from these SPR and semiconductor-based photocatalysts, organic materials such as g-C_3_N_4_ and graphene are recently experiencing a burgeoning period in photoredox organic synthesis (Nan et al., 2013; Yang and Xu, 2013; Zhang et al., 2015). A number of transformations such as C-N, C=N formation, alcohol oxidation to carbonyl, nitroarene reduction to aniline, N = N formation in azobenzene, etc., were successfully achieved by these carbon-based materials (Su et al., 2010; Dai et al., 2018). In spite of some disadvantages such as limited substrate scopes and reaction types, less functional group tolerability and poor chemo- and regioselectivity, TiO_2_ photocatalysis has its incomparable long-comings: being extremely stable under strong acidic, basic, oxidative, reductive and illuminative conditions, non-toxic to environment, facile in recycling, and reusing without apparent loss of catalytic activity. TiO_2_ photocatalysis is considered as the future star for applications in organic synthesis. To cover the current gaps, more research focus should be concentrated on the following several issues: (i) Enlarging the scope of TiO_2_ absorption is very important for more efficient transformations initiated by visible-light or even near-infrared light. (ii) Developing TiO_2_ photocatalysis systems with stereoselectivity can meet the demand for contemporary medicinal chemistry. (iii) More reaction types should be established and wider substrates scopes and better functional group tolerability should be achieved. (iv) The gram-scale or kilogram-scale synthesis by TiO_2_ photocatalysis should be emphasized via promoting the quantum efficiency of photocatalysts or quickly removing the products from the surface of photocatalysts. Thereby, TiO_2_ photocatalysis will embrace a brighter future in the field of natural product, pharmaceuticals, agrochemicals, and fine-chemicals synthesis after these demands are met.

## Author Contributions

DM designed this proposal, determined the contents, wrote the Abstract, Introduction, TiO_2_ Photocatalyzed Amine N-alkylation and Conclusion. SZ wrote the TiO_2_ photocatalyzed N-heterocycle formation. AL and YW drew the figures. CC revised the manuscript. All authors contributed for the writing of the manuscript.

### Conflict of Interest Statement

The authors declare that the research was conducted in the absence of any commercial or financial relationships that could be construed as a potential conflict of interest.
